# Rewilding catalyzes maturation of the humoral immune system

**DOI:** 10.1126/sciadv.ads2364

**Published:** 2025-03-07

**Authors:** Ying-Han Chen, Kimberly Zaldana, Frank Yeung, Ivan Vujkovic-Cvijin, Alexander E. Downie, Jian-Da Lin, Yi Yang, Christin Herrmann, Oyebola Oyesola, Felix Rozenberg, Robert E. Schwartz, David Kim, Kurt Tio, Yasmine Belkaid, P’ng Loke, Andrea L. Graham, Sergei B. Koralov, Ken Cadwell

**Affiliations:** ^1^ Institute of Biomedical Sciences, Academia Sinica, Taipei, Taiwan.; ^2^ Vilcek Institute of Graduate Biomedical Sciences, New York University Grossman School of Medicine, New York, NY 10016, USA.; ^3^ Department of Pathology, New York University Grossman School of Medicine, New York, NY 10016, USA.; ^4^ Department of Biomedical Sciences & F. Widjaja Inflammatory Bowel Disease Institute, Cedars-Sinai Medical Center, Los Angeles, CA 90048, USA.; ^5^ Department of Primate Behavior and Evolution, Max Planck Institute for Evolutionary Anthropology, Leipzig, Germany.; ^6^ Department of Biochemical Science and Technology, College of Life Science, National Taiwan University, Taipei City, Taiwan.; ^7^ Division of Gastroenterology and Hepatology, Department of Medicine, University of Pennsylvania Perelman School of Medicine, Philadelphia, PA 19104, USA.; ^8^ Laboratory of Parasitic Diseases, National Institute of Allergy and Infectious Diseases, National Institutes of Health, Bethesda, MD 20892, USA.; ^9^ SUNY Downstate Health Sciences University, New York, NY 11203, USA.; ^10^ Division of Gastroenterology and Hepatology, Department of Medicine, Weill Cornell Medicine, New York, NY 10065, USA.; ^11^ Laboratory of Host Immunity and Microbiome, National Institute of Allergy and Infectious Diseases, National Institutes of Health, Bethesda, MD 20892, USA.; ^12^ Metaorganism Laboratory, Department of Immunology, Pasteur Institute, Paris, France.; ^13^ Department of Ecology and Evolutionary Biology, Princeton University, Princeton, NJ 08544, USA.; ^14^ Santa Fe Institute, Santa Fe, NM 87501, USA.; ^15^ Department of Pathobiology, University of Pennsylvania School of Veterinary Medicine, Philadelphia, PA 19104, USA.

## Abstract

Inbred mice used for biomedical research display an underdeveloped immune system compared with adult humans, which is attributed in part to the artificial laboratory environment. Despite representing a central component of adaptive immunity, the impact of the laboratory environment on the B cell compartment has not been investigated in detail. Here, we performed an in-depth examination of B cells following rewilding, the controlled release of inbred laboratory mice into an outdoor enclosure. In rewilded mice, we observed B cells in circulation with increased signs of maturation, alongside heightened germinal center responses within secondary lymphoid organs. Rewilding also expanded B cells in the gut, which was accompanied by elevated systemic levels of immunoglobulin G (IgG) and IgM antibodies reactive to the microbiota. Our findings indicate that exposing laboratory mice to a more natural environment enhances B cell development to better reflect the immune system of free-living mammals.

## Teaser

Releasing laboratory mice into the natural environment enhanced maturation and activation of the humoral immune system.

## INTRODUCTION

Confined to cages within specific pathogen–free (SPF) facilities, laboratory mice are shielded from events that are commonly experienced by free-living mammals such as infections, complex diets, social interactions, and fluctuations in weather. This isolation from environmental variables profoundly affects the immune system, potentially explaining the discrepancies observed between laboratory mice with humans (*1*–*3*). Supporting the pivotal role of microbes in this disparity, the immune system of laboratory mice evolves toward a state similar to that of an adult human when exposed to sequential infections by pathogens or cohousing with “dirty” mice carrying infectious agents (*4*–*8*). Inbred laboratory mice reconstituted with microbiota from wild mice through fecal microbiome transplantation or vertical transmission more accurately model disease processes and drug responsiveness in humans (*9*–*12*). In addition, laboratory mice cohoused with pet store mice, which leads to transmission of pathogenic agents, exhibit altered vaccine-induced humoral responses that closely resemble the responses observed in humans (*13*). These observations highlight the importance of defining the impact of environmental exposure on immune cells, including the B cell compartment.

To address these challenges, we established a mesocosm system known as rewilding, in which laboratory mice are exposed to a seminatural environment through controlled release into an outdoor enclosure facility (*14*). Interactions with this environment (e.g., soil, insects, and vegetation) facilitate exposure to microbes found in nature, but wild rodents are excluded. Thus, rewilded mice are generally not exposed to disease-causing pathogens found in other dirty mouse models, offering a unique approach to studying environmental effects without pathogenic confounders (*15*). Rewilded laboratory mice displayed several hallmarks of increased immune activation, including a higher proportion of differentiated memory T cells and the expansion of circulating granulocytes (*15*–*17*). Further analysis of flow cytometry data indicated that CD44hiCD19^+^ lymphocytes are among the most enriched cellular subsets in the blood of rewilded mice compared with laboratory controls (*16*). Because these markers are associated with activated and memory B cells (*18*), this observation raises the possibility that exposure to a natural environment contributes to the maturation of the humoral immune system, which we examined here.

## RESULTS AND DISCUSSION

**Fig. 1.**
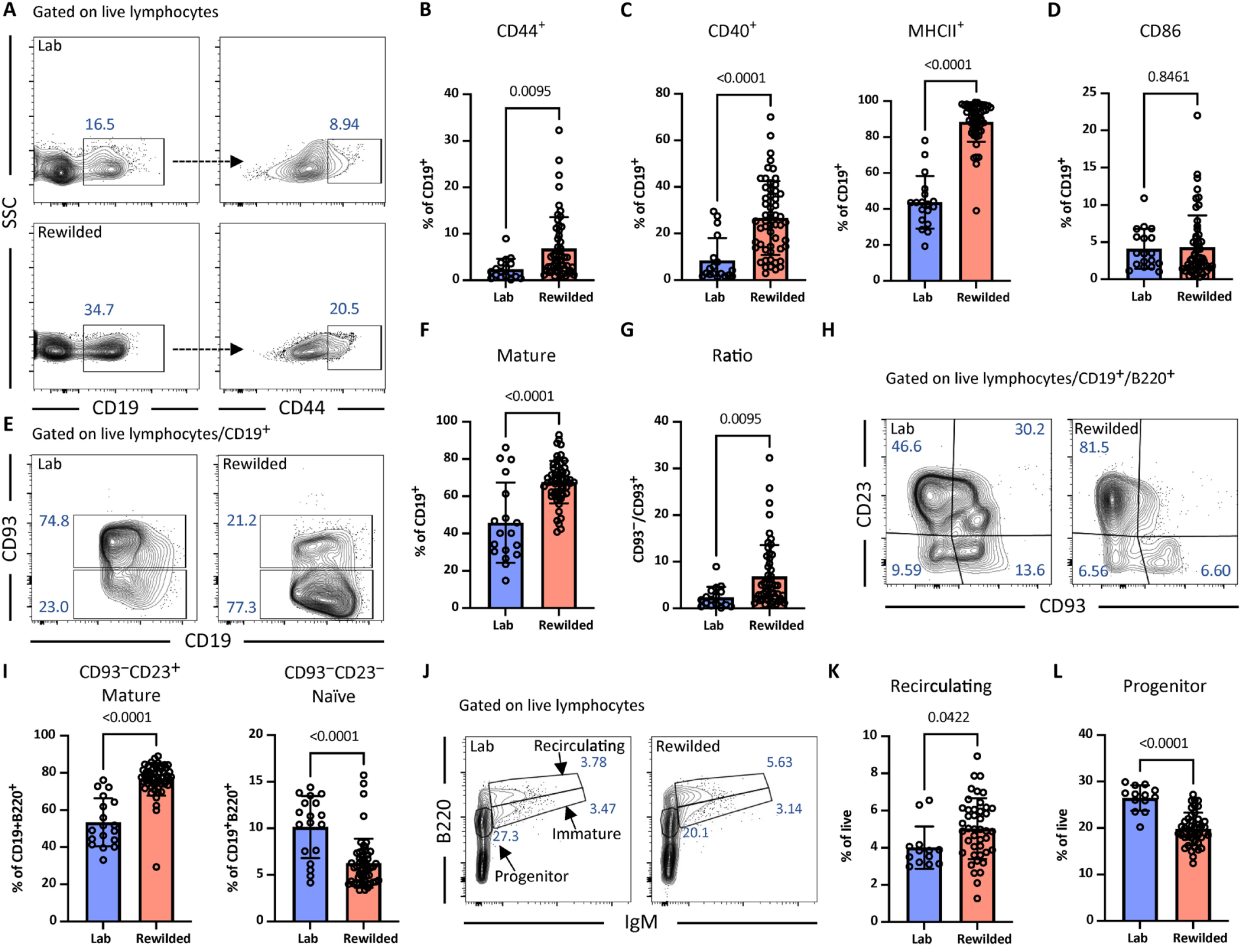
Rewilding increases the maturation of B-lymphocytes. (**A**) Representative flow cytometry plots depicting gating strategy for CD44^+^ B cells in peripheral blood from laboratory (Lab) and rewilded wild-type (*n* = 5 laboratory and 18 rewilded), *Dectin-1^−/−^* (*n* = 4 laboratory and 10 rewilded), *Dectin-1^+/−^* (*n* = 5 laboratory and 10 rewilded), *Card9^−/−^* (*n* = 3 laboratory and 8 rewilded), and *Card9^+/−^* (*n* = 8 rewilded) mice. B cells were gated from live lymphocytes by FSC/SSC (forward scatter/side scatter) dot plot and characterized as CD19^+^ cells. (**B** to **D**) Quantification of frequency of CD44^+^, CD40^+^, MHCII^+^, and CD86^+^ B cells from (A). (**E**) Representative flow cytometry plots depicting gating strategy used to identify mature B cells as CD93^−^ fraction and immature B cells as CD93^+^ fraction in peripheral blood of both laboratory and rewilded mice. (**F**) Bar graph depicting frequency of CD93^−^ mature B cells from (E). (**G**) Ratio of CD93^−^ fraction to CD93^+^ from B cells in peripheral blood. (**H**) Representative flow cytometry plots depicting gating strategy for CD93^−^CD23^+^ mature and CD93^−^CD23^−^ naïve B cell subsets in peripheral blood from laboratory and rewilded mice. (**I**) Quantification of frequency of B cell subsets in peripheral blood from CD19^+^B220^+^ B cells in (H). (**J**) Representative flow cytometry plots depicting gating strategy used to identify recirculating, immature, progenitor B cells in the BM from laboratory and rewilded wild-type (*n* = 5 laboratory and 9 rewilded), *Dectin-1^−/−^* (*n* = 4 laboratory and 10 rewilded), *Dectin-1^+/−^* (*n* = 10 rewilded), *Card9^−/−^* (*n* = 4 laboratory and 8 rewilded), and *Card9^+/−^* (*n* = 6 rewilded) mice. Recirculating and immature B cells were gated from live lymphocytes and characterized as B220hiIgM^+^ and B220loIgM^+^, respectively. (**K** and **L**) Frequency of recirculating (B220hiIgM^+^) and progenitor (B220+IgM^−^) B cells in BM. Dots in bar graphs correspond to individual mice. Means and SD are shown. Indicated *P* values by two-tailed Student’s *t* test between groups.

In an independent rewilding experiment to confirm and expand upon prior findings, 6- to 8-week-old SPF laboratory C57BL6/J mice were released into the enclosure and captured 6 weeks later, at which point B cell subsets were compared with littermate controls housed in the institutional animal facility through flow cytometry analysis of surface markers (fig. S1A). We first examined the circulating B cells in the peripheral blood and confirmed that the expansion of CD44hiCD19^+^ B cells that occurs upon rewilding was reproducible despite the 2-year gap between the experiments (Fig. 1, A and B). In addition, we observed an increase in B cell activation markers CD40 and major histocompatibility complex II (MHCII), but not CD86 from the cohort of rewilded mice (Fig. 1, C and D, and fig. S1, B to F). Although the immune system of laboratory mice remains immature through adulthood, immature B lymphocytes gradually decline from birth to 16 years of age in humans (*19*). To assess whether B cell maturation in laboratory mice is enhanced after exposure to the natural environment, we used CD93, also known as complement component C1q receptor (C1qRp) or AA4.1, as a marker to identify transitional B cells, representing a stage of B cell development between immature and mature B cells (*20*). Compared with laboratory mice, rewilded mice exhibited a decrease in CD93^+^CD19^+^ B cells and a corresponding rise in CD93^−^CD19^+^ mature B cells in the peripheral blood, leading to an increased ratio of CD93^−^ to CD93^+^ cell (Fig. 1, E to G, and fig. S1, G and H). Among CD93^−^CD19^+^B220^+^ mature B cells, the CD23^−^ naive B cell population was reduced in rewilded mice; this decrease in CD23^−^ naive B cells was associated with an increase in CD23^+^ mature B cells, consistent with an accelerated maturation process (Fig. 1, H and I).

Although we observed a reduction in the proportion of B cells in the bone marrow (BM) (fig. S1I), a primary site for B cell development and maturation, rewilding led to increases in B220hiIgM^+^ recirculating mature B cells and ratio of these recirculating mature B cells to immature B cells (Fig. 1, J to I, and fig. S1, J and K). The proportion of B-lineage progenitors decreased because of the expansion of myeloid lineage cells in the BM (*17*); however, the proportions of pro B and pre B cells remained unchanged when comparing laboratory and rewilded mice (fig. S1, L to N). These results suggest that rewilding is associated with a general acceleration in B cell maturation and activation.

**Fig. 2.**
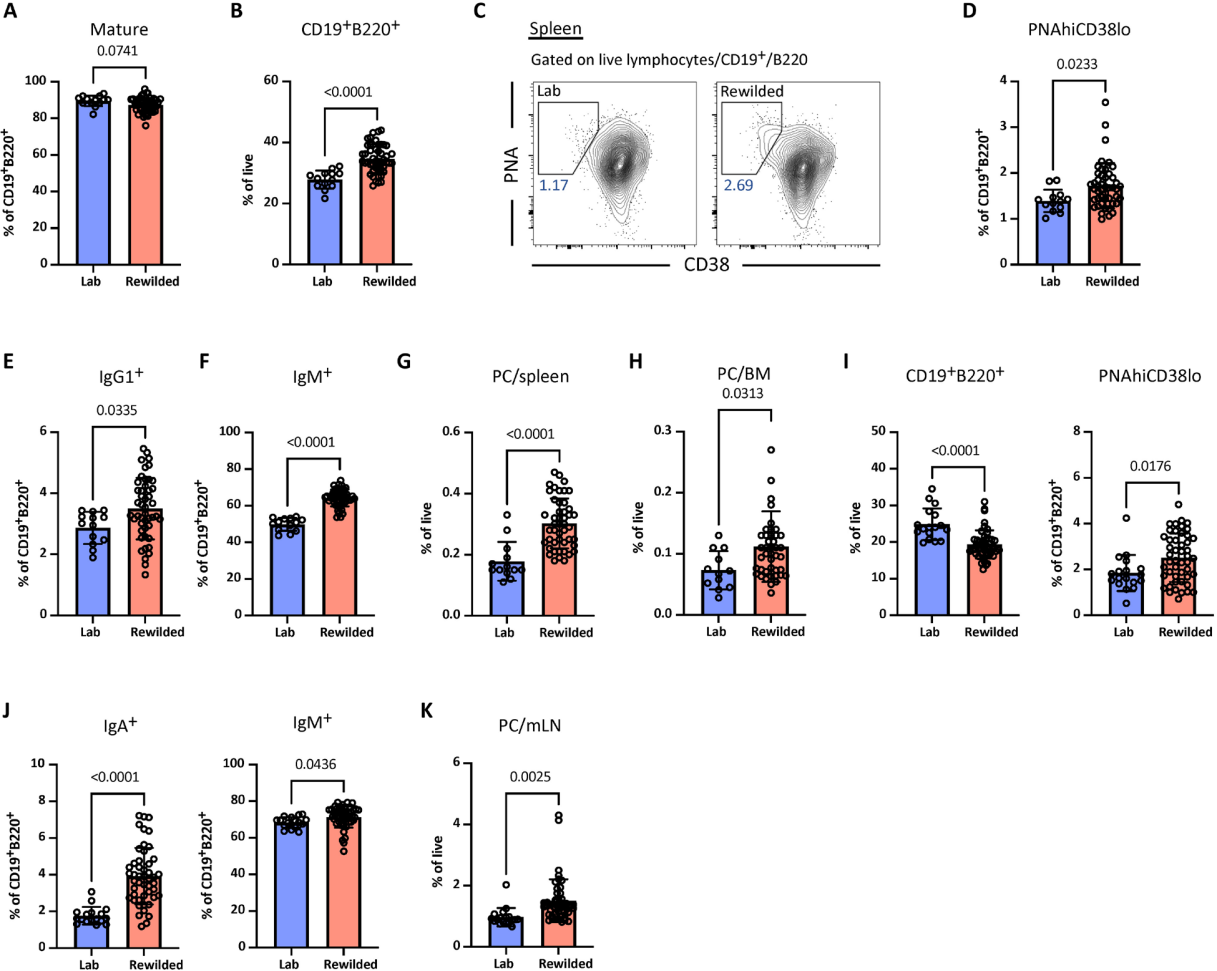
Rewilding promotes GC responses. (**A**) Bar graph depicting frequency of mature B cells in the spleen of laboratory and rewilded mice. (**B**) Frequency of B cells (CD19^+^B220^+^) in the spleen of laboratory and rewilded wild-type (*n* = 5 laboratory and 11 rewilded), *Dectin-1^−/−^* (*n* = 4 laboratory and 10 rewilded), *Dectin-1^+/−^* (*n* = 10 rewilded), *Card9^−/−^* (*n* = 4 laboratory and 8 rewilded), and *Card9^+/−^* (*n* = 8 rewilded) mice. (**C**) Representative flow cytometry plots depicting gating strategy for GC B cells in the spleen from laboratory and rewilded mice. GC B cells were gated on CD19^+^B220^+^ B cells and identified by CD38 and peanut agglutinin (PNA). (**D**) Quantification of frequency of PNAhiCD38lo GC B cells from (C). (**E** and **F**) Frequency of B cells expressing IgG1 and IgM in the total number of CD19^+^B220^+^ B cells sampled from the spleen. (**G** and **H**) Frequency of plasma cells (CD138^+^) in the spleen and BM. (**I**) Quantification of total B cells and PNAhiCD38lo GC B cells in the mLN from laboratory and rewilded wild-type (*n* = 5 laboratory and 15 rewilded), *Dectin-1^−/−^* (*n* = 4 laboratory and 8 rewilded), *Dectin-1^+/−^* (*n* = 5 laboratory and 10 rewilded), *Card9^−/−^* (*n* = 4 laboratory and 7 rewilded), and *Card9^+/−^* (*n* = 8 rewilded) mice. (**J**) Frequency of IgA^+^ and IgM^+^ B cells. (**K**) Bar graph depicting frequency of plasma cells in the mLN. Dots in bar graphs correspond to individual mice. Means and SD are shown. Indicated *P* values by two-tailed Student’s *t* test between groups.

To assess whether exposure to the natural environment enhances the germinal center (GC) response, which is essential for affinity maturation and high-affinity antibody production, we analyzed B cell populations from the spleen and mesenteric lymph nodes (mLNs). As expected, the vast majority of B cells in the spleen of rewilded and laboratory control mice were mature (CD93^−^) (Fig. 2A and fig. S2A). Furthermore, rewilded mice displayed an increase in the proportion of total B cells (B220^+^CD19^+^) compared with laboratory controls (Fig. 2B). CD40 signaling in B cells promotes GC formation and immunoglobulin (Ig) isotype switching (*21*). Consistent with the increase in circulating CD40^+^ B cells, we noted a marked expansion of GC B cells and class-switched IgG1^+^ B cells in the spleen of rewilded mice compared with laboratory mice without affecting the proportion of marginal zone and follicular B cells (Fig. 2, C to E, and fig. S2, B and C). However, IgM^+^ B cells still dominated the spleen of rewilded mice (Fig. 2F). Plasma cells, terminally differentiated B cells that secrete high levels of antibodies, increased following rewilding (Fig. 2G and fig. S2D). A fraction of the plasma cells migrate to the BM, where they become resident long-lived plasma cells (*22*). We also observed an increase in the population of plasma cells within the BM (Fig. 2H and fig. S2E).

The gastrointestinal tract serves as an interface between the immune system and dietary and microbial antigens derived from the external environment. Thus, we examined the B cell profile of the mLN, which drains intestinal organs. In contrast to the spleen, there was a modest reduction in total B cells (B220^+^CD19^+^) following rewilding. However, there was a notable proportional expansion of GC B cells and increases in both IgA^+^ and IgM^+^ B cells in the mLN of rewilded mice (Fig. 2, I and J, and fig. S2F). Similar to the spleen and BM, the mLN of rewilded mice displayed a higher abundance of plasma cells compared to mice in the laboratory setting (Fig. 2K). Overall, these findings illustrate that rewilding induces enhanced GC responses and terminal differentiation of B cells into antibody-producing plasma cells.

**Fig. 3.**
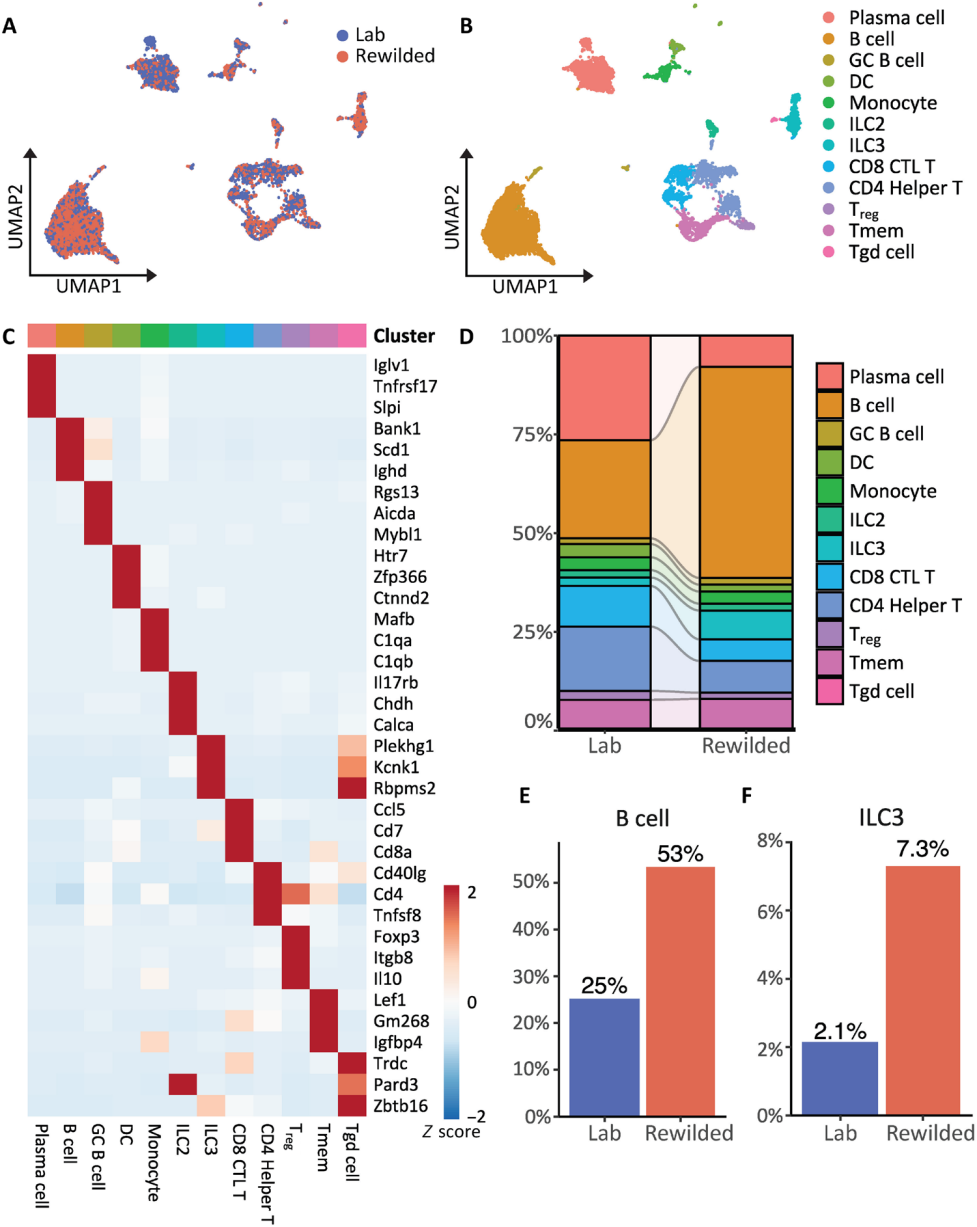
Rewilding leads to an expansion in intestinal B cells. (**A** and **B**) Uniform Manifold Approximation and Projection (UMAP) visualization of scRNA-seq data from CD45^+^ cells isolated from the small intestine comparing laboratory and rewilded mice and assignment of immune cell identity to each identified clusters using ImmGenData reference database. *N* = 4 laboratory and 4 rewilded mice. (**C**) Heatmap representing the top three most highly expressed genes within each cluster relative to the rest of the clusters. (**D**) Bar plot of the proportion of cells in laboratory and rewilded mice for each cell cluster from (B). (**E** and **F**) Proportion of B cells and ILC3 cells in the small intestine of laboratory and rewilded mice.

The above analyses focused on primary and secondary lymphoid organs. To delve deeper into the immune landscape of the gut, we used single-cell RNA sequencing (scRNA-seq) of CD45^+^ cells sorted from the intestinal lamina propria of laboratory and rewilded mice. We used unsupervised dimensionality reduction and unbiased cell type recognition using the ImmGenData open-source reference database for gene expression to identify immune cell populations. Each cluster was distinguished by the expression of well-defined marker genes and linked to a distinct cell type or state, encompassing dendritic cells, monocytes, innate lymphoid cells (ILCs), and B and T lymphocytes (Fig. 3, A to C, and fig. S3A). Within the T cell compartment, we identified cytotoxic CD8 T cells (CD8 CTL T), helper CD4 T cells (CD4 helper T), gamma-delta T cells (Tgd), regulatory T cells (T_regs_), and memory T cells (Tmem). In the B cell compartment, we identified three distinct clusters: CD19^+^ B cells (B cells), GC B cells, and plasma cells. The representation of cell clusters was distinct for rewilded and laboratory mice. For instance, rewilded mice displayed a notable increase in the proportion of B cells within the small intestine, accompanied by an expansion of type 3 ILCs (ILC3s) (Fig. 3, D to F, and fig. S3B), which have been shown to regulate B cell responses in both the intestine and spleen (*23*, *24*). Up-regulation of ILC3s and associated interleukin-17 (IL-17)^+^ CD4 T cells can occur upon colonization by adherent commensal bacteria and fungi (*25*, *26*) and is consistent with our previous finding that rewilding leads to enhanced IL-17 secretion in restimulated mLN cells (*15*).

**Fig. 4.**
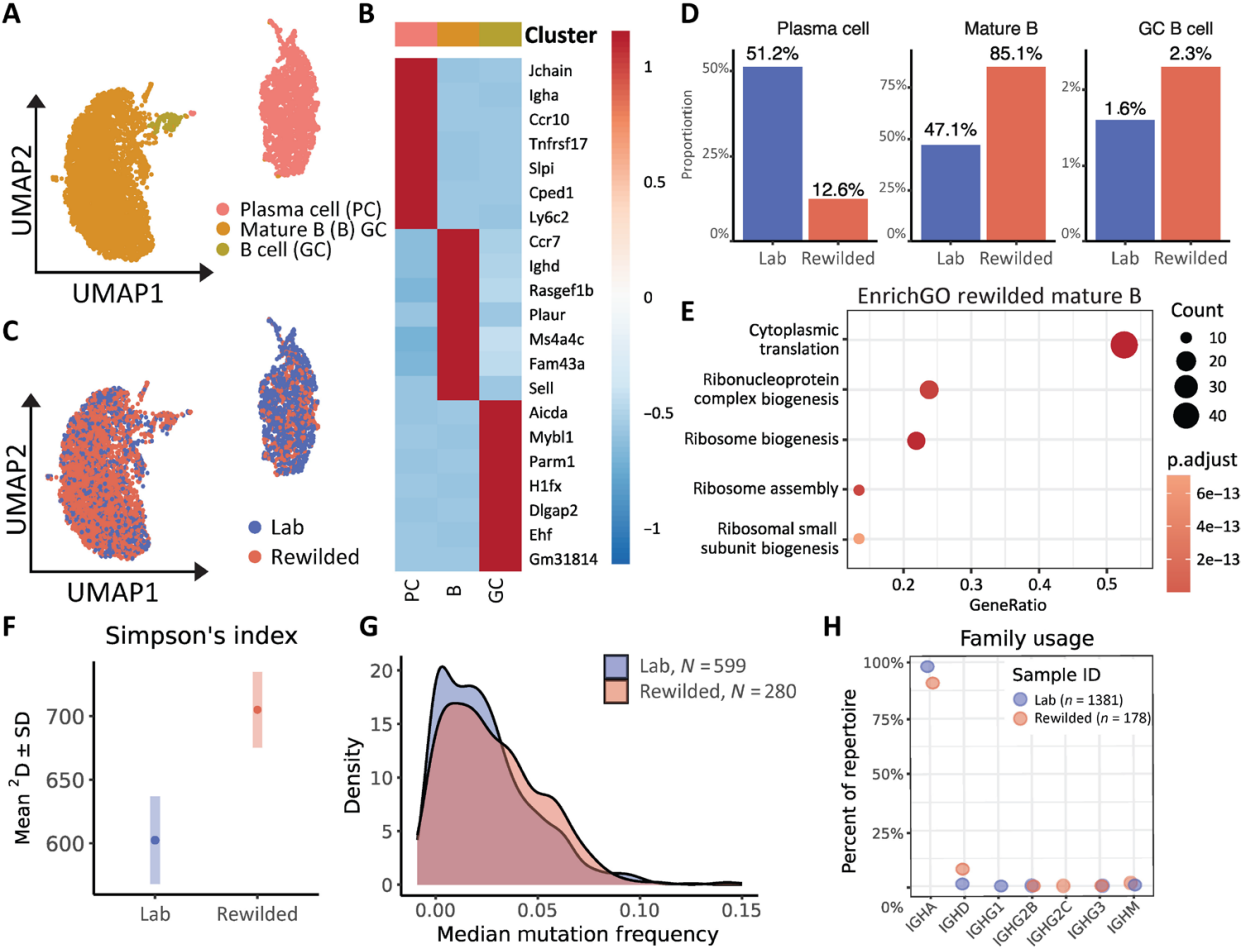
Rewilding leads to expansion of mature naïve and activated B cells in the gut. (**A**) Unsupervised reclustering of B-lineage subsets from Fig. 3A and assignment of each B cell identity. (**B**) Heatmap representing the highly expressed genes within each B cell cluster relative to the rest of the clusters. (**C**) UMAP of B-lineage clusters from Fig. 3A and comparison of the clusters by UMAP plot between laboratory and rewilded mice. (**D**) Bar plot of the proportion of cells in laboratory and rewilded mice for each B cell cluster identified. (**E**) Gene ontology enrichment analysis of the differentiated genes from mature B cells of rewilded mice. (**F**) Alpha diversity analysis using Simpson’s Index of unique BCRs between laboratory and rewilded mice. (**G**) Median mutational frequency in plasma cell clones between laboratory and rewilded mice. (**H**) Constant region usage frequency between laboratory and rewilded mice.

Next, we zeroed in on B-lineage cells within our analyzed events in Fig. 3B because B cells in mucosal tissue, especially the gut, have received less attention in dirty mouse models compared with other compartments. The B cells separated into three clusters, which corresponded to mature B cells, GC B cells, and plasma cells (Fig. 4, A to C, and fig. S4A). Consistent with our flow cytometric analysis that highlighted increases in mature B lymphocytes, our scRNA-seq analysis of intestinal lymphocytes revealed mature B cell populations, enriched for *Ighd* (IgD), *Cd22* (CD22), *Cr2* (complement receptor 2), and *Fcer2a* (CD23) expression, were increased in rewilded mice compared with their counterparts in the laboratory setting (Fig. 4D and fig. S4C). Gene ontology enrichment analysis of the differentiated genes from mature B cells suggested a distinct metabolic profile in rewilded mice compared to laboratory mice, characterized by enhanced cytoplasmic translation, oxidative phosphorylation, aerobic respiration, purine ribonucleoside triphosphate biosynthetic processes, and proton motive force–driven adenosine triphosphate synthesis (Fig. 4E and fig. S4B). This observation is consistent with the increased demand on mitochondrial respiration required during B cell activation (*27*, *28*).

We did not observe an increase in the relative abundance of plasma cells by scRNA-seq because, as a proportion of all B cells, plasma cells were overshadowed by the dramatic increase in the mature B cells. We isolated a substantially higher number of CD45^+^ cells from rewilded mice, suggesting that the total number of plasma cells may actually be increased. Therefore, we performed immunostaining with the plasma cell marker CD138, which revealed a robust increase of CD138^+^ cells in rewilded mice compared with laboratory controls (fig. S4D). These findings indicate that rewilding leads to notable differences in intestinal B cell maturation, activation, differentiation, and metabolism, identifying an expansion of mature B cells in the immune system of mice exposed to the natural environment compared with laboratory mice.

Our findings revealed increased B cell activation in peripheral tissues and notable changes in B cell metabolism within the small intestine. These observations underscore the importance of further exploring how heightened activation and metabolic alterations contribute to the broader role of B cells in immune function. The connection between metabolism and B cell function emerged from our investigation into how transcriptional differences in metabolic pathways might affect B cell receptor (BCR) repertoire and functionality. Consequently, we aimed to evaluate the impact of rewilding on the BCR repertoire, which could have substantial implications for humoral immunity by examining B cell diversity and somatic hypermutations. Leveraging de novo assembly, we reconstructed the sequence of single-cell BCRs and inferred clonality using Trust4. Subsequently, we conducted an in-depth analysis of BCR diversity, somatic hypermutation, gene usage, and clonal evolution of all reconstructed BCRs with unique identifiers, using Immcantation. We successfully captured 1381 and 1178 productive BCRs in laboratory and rewilded mice, respectively, as depicted in the network analysis based on similar clones among receptor sequences (fig. S5, A and B). Clonal assignments and lineage reconstruction were facilitated by hierarchical clustering, enabling us to track point somatic hypermutations in clonal evolution, illustrated by example trees and sequences (fig. S5C). Alpha diversity analysis revealed an increase in Richness and Simpson’s Index, suggesting a greater diversity of unique BCRs that are more evenly distributed in rewilded mice (Fig. 4F). We then assessed the median mutational frequency in plasma cell clones, which presumably underwent maturation in a GC. Plasma cell clones in rewilded mice exhibited a slight increase in median mutational frequency compared to laboratory mice, as indicated by the rightward shift of the curve and supported by the Kolmogorov-Smirnov test’s *P* value of 0.02675 and *D* value of 0.10633 (Fig. 4G). When comparing constant region usage, rewilded mice demonstrated an increase in IGHD, aligning with the increase of mature IgD^+^ B cells identified in the scRNA-seq (Fig. 4H). In conclusion, our analysis of single-cell BCRs, clonal evolution, and mutational frequency, coupled with our findings on the increased IGHD in rewilded mice, suggests a more diverse and mature B cell response in rewilded mice compared to laboratory mice.

**Fig. 5.**
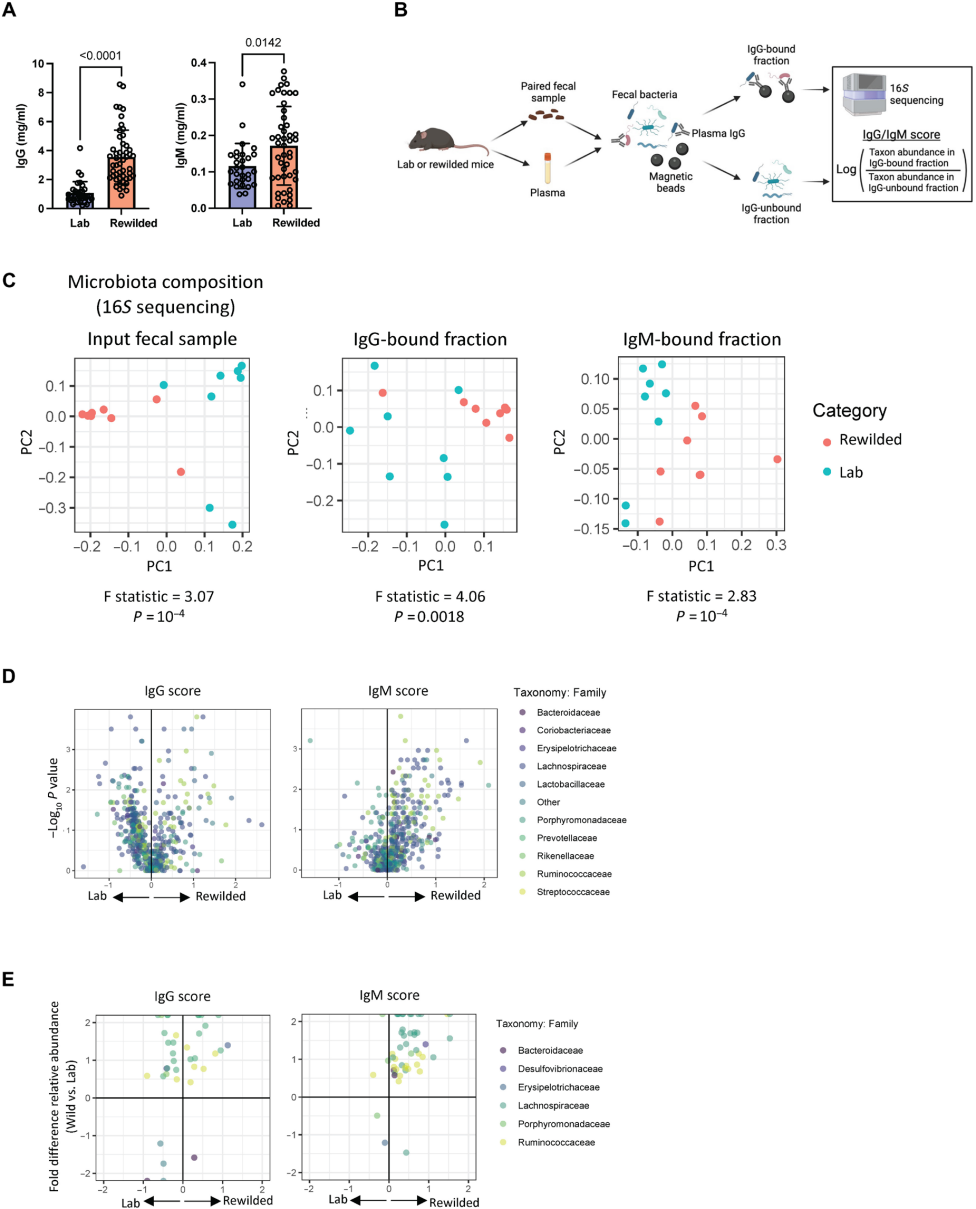
Increased circulating IgG and IgM in rewilded mice is associated with alterations in the gut microbiota. (**A**) Quantification of IgG and IgM in the blood from laboratory and rewilded mice. (**B**) Schematic of the IgG- and IgM-seq workflow. Fecal bacteria from laboratory or rewilded mice were incubated with autologous plasma and stained with anti-IgG or anti-IgM magnetic beads antibodies. The fractions were subjected to 16*S* rRNA sequencing, and IgG and IgM scores for each taxon were calculated as the log ratio of taxon relative abundance in the IgG- and IgM-bound fraction over that in the unbound fraction. *N* = 8 laboratory and 8 rewilded mice. (**C**) PCoA of input microbes, IgG- and IgM-bound fraction using pairwise Pearson correlation coefficient matrices encompassing IgG scores for all taxa are shown. *P* values and *F* statistics were calculated using PERMANOVA. (**D**) Mean fold differences in IgG and IgM scores (*x* axis) and *P* values determined by Mann-Whitney *U* tests (*y* axis) for each antibody-bound taxa comparing rewilded and laboratory mice. Family level identity of the taxa indicated in the legend. (**E**) Scatter plots of relative abundance of the indicated taxa in the total microbiota (*y* axis) versus corresponding IgG and IgM scores (*x* axis) comparing rewilded and laboratory mice.

Given the increased B cell activation across various compartments and BCR repertoire diversification, we asked whether there was a corresponding amplification of antibody production and specificity toward microbes. We collected plasma samples from individual laboratory and rewilded mice, assessing the systemic levels of total IgG and IgM, the two Ig classes predominantly found in the peripheral blood. Rewilded mice exhibited elevated levels of both IgG and IgM compared with laboratory mice (Fig. 5A). Increased IgG and IgM levels often accompany a response to an infection or exposure to foreign antigens that breach a mucosal barrier. Consistent with our prior finding, a comprehensive serology panel showed that rewilded mice tested negative for all pathogens that were analyzed (table S1). Through a polymerase chain reaction (PCR)–based analysis of fecal samples, we detected a few opportunistic pathogens, such as *Klebsiella* species and *Staphylococcus aureus*, in a subset of rewilded mice (table S2). These microbes are found in SPF facilities and frequently described as commensals. Thus, we shifted our focus to the microbiota as a potential target of the humoral immune system.

We were particularly interested in the increased levels of circulating IgG and IgM in rewilded mice (Fig. 5A) that matched the increased numbers of IgG^+^ and IgM^+^ B cells in the spleen (Fig. 2, E and F), a filtering organ. In contrast to mucosal IgA, serum IgG preferentially binds translocating microbes (*29*). Thus, we investigated whether plasma IgG and IgM targeted different gut bacteria in laboratory versus rewilded mice. First, we performed 16*S* ribosomal RNA (rRNA) sequencing of stool samples for an overview of the gut microbiome structure, which indicated that rewilding was associated with enrichment of *Clostridiales* and a decrease in *Erysipelotrichales* species (fig. S6, A and B). Next, we identified IgG- and IgM-bound taxa by incubating plasma samples with matching autologous fecal samples (Fig. 5B). Fecal samples are enriched in IgA, precluding a similar approach to determine reactivity of plasma IgA (*30*). The overall microbiome composition (input) as well as the IgG- and IgM-bound fractions was distinct in rewilded compared with laboratory mice according to principal coordinates analysis (PCoA) (Fig. 5C). We then calculated IgG and IgM scores as log ratios of taxon abundances in the bound versus unbound fractions (*29*), which showed that for both antibody classes, the bacteria that were targeted were notably distinct in rewilded mice compared with laboratory controls (Fig. 5D). As an example, *Lactobacillus* species were increased among the IgG-bound fractions in rewilded mice, while *Akkermansia muciniphila* was reduced (fig. S6, C and D).

IgM scores, which reflect enrichment of a given IgM-bound bacterial taxon over the unbound fraction, were higher in rewilded mice across a multitude of taxa compared to laboratory mice (Fig. 5D). Notably, the taxa with increased IgM binding in rewilded mice were also enriched in the total microbiota of rewilded mice compared to their laboratory counterparts (Fig. 5E). This observation suggests that the heightened IgM levels observed in rewilded mice represent a response to newly acquired gut bacteria during the rewilding process. In contrast, the increase in IgG binding observed in rewilded mice does not align with changes in the abundance of bacterial taxa, suggesting that elevated IgG levels were driven by immune modulation mechanisms independent of specific bacteria. As an example, rewilding conditions may alter intestinal barrier permeability and modify antigen presenting cells along with CD4^+^ T cells, potentially changing IgG responses without affecting bacterial populations that serve as antigen sources. Alternatively, the increase in IgG may reflect a response to a nonbacterial antigen or a transient exposure to a microbe not detected at the terminal time point. These findings indicate that, although IgM responses were directly tied to changes in microbiota composition, IgG responses may be influenced by different aspects of the rewilded environment, reflecting a more complex aspect of immune adaptation in these mice.

Together, our findings demonstrate that exposure to a more natural environment induces enhanced maturation and activation of B cells associated with an expansion of GC B cells in secondary lymphoid organs. Rewilded mice concurrently displayed an increase in IgG1^+^ and IgA^+^ B cells in the spleen and mLNs, respectively. Consistent with the gastrointestinal tract as an interface with the environment, we observed an elevated presence of mature B cells and plasma cells in the gut of rewilded mice. Notably, changes in rewilded mature B cell metabolism is interesting given that recent studies have suggested that metabolic reprogramming plays a critical role in orchestrating B cell differentiation and function (*28*). During B cell activation, there is a shift toward increased glycolysis and oxidative phosphorylation to meet energy and biosynthetic demands (*31*). In the future, it would be interesting to investigate whether these metabolic changes in rewilded B cells also affect their development and differentiation into plasma cells and their other effector functions such as antigen presentation and cytokine secretion. In addition, heightened antibody levels, especially IgM, in rewilded mice were associated with changes in the gut microbiota that occurred upon exposure to the natural environment. Because of the limitations of sample size, we were unable to validate whether rewilding influences specific IgG subclass responses. Investigating IgG subclass responses will be an important focus for future studies. In addition, reconstructed BCRs in our scRNA-seq data reveal enhanced IgD expression, which aligns with our observation of increased mature B cells in the periphery. While we observe some variations in IgG subclass usage between rewilded and laboratory mice, the limited capture depth of our dataset prevents us from making definitive claims about individual IgG subclass distributions. It remains unclear how these changes to the B cell compartment at homeostasis would affect immunity to pathogens or vaccination. Mice cohoused with pet store mice display a dampened humoral response to influenza in response to a vaccine (*13*). It is possible that a heightened state of B cell maturation upon exposure to the natural environment interferes with nonmucosal vaccine responses, or, alternatively, the pathogens acquired upon cohousing with pet store mice cause a fundamentally different effect on B cell development. A careful cross-comparison of B cells and their functions across platforms is warranted.

Our findings underscore the impact of microbes and likely other environmental variables on the B cell compartment. An important future direction would be to identify specific microbes that drive the individual changes to the B cell compartment. For example, we previously showed that rewilding leads to heighted fungal exposure in the gut that can increase circulating neutrophils. Although the low number of fungal genomes relative to bacteria did not allow us to measure IgG and IgM binding, fungal species have been shown to activate B cells via pattern recognition receptors like Toll-like receptor 2, leading to increased production of antibodies, particularly IgG1 and IgM (*32*). Laboratory mice have a paucity of memory B cells and less diverse BCRs compared with humans (*33*). Given that rewilded mice display increases in maturation and activation markers consistent with an expansion in memory B cells, our results suggest that previously observed differences between mice and humans may be, at least in part, attributed to the absence of such variables in laboratory conditions. Hence, rewilding and other approaches that expose laboratory mice to environmental variables may be particularly useful for investigating B cell–dependent processes.

## METHODS

### Study design

The objective of this study was to elucidate how exposure to a natural environment influences the maturation of the humoral immune system. B cell populations in peripheral blood, BM, spleen, and mLNs were analyzed using flow cytometry to compare differences between laboratory and rewilded mice. To provide a comprehensive understanding of immune cell populations in the gut following rewilding, scRNA-seq analysis was conducted on immune cells from the lamina propria of the small intestine to identify expanded cell subsets. In addition, we evaluated the systemic anti-microbiota IgG and IgM repertoire, considering the microbiota as a potential target of the humoral immune system. Mice were randomly assigned to individual groups, and outliers were retained in the analysis. Details of the rewilding procedure and facility are provided below, and figure legends include information on the number of mice and statistical analyses used in the studies.

### Mice and outdoor enclosure

This study analyzed specimens that were collected from rewilded mice described in a companion study (*17*). Mice on the C57BL/6J background were purchased from the Jackson Laboratory and then bred onsite in an murine norovirus (MNV)/Helicobacter-free SPF facility at NYU Grossman School of Medicine. The cohort of mouse genotypes included C57BL/6J [wild type (WT)], Dectin-1^−/−^ (Clec7a^−/−^), Dectin-1^+/−^, and Card9^−/−^ (*17*). Littermates from breeding pairs were randomly assigned to either remain in the institutional vivarium (laboratory mice) or be released into outdoor enclosures (rewilded mice) to ensure consistent microbiota conditions at the start of the experiment. In the previous study, both male and female mice were analyzed and the impact of sex on immune variables was shown to be minimal and not significant for lymphocyte numbers (*16*, *17*). To prevent breeding and aggressive behavior in the outdoor enclosure, only female mice were used for rewilding in the subsequent experiment while both sexes were included as laboratory controls.

The outdoor enclosures, previously described, consist of wedges, each about 180 m^2^ in size, fenced by 1.5-m-high zinced iron walls buried >80 cm deep and topped with electrical fencing to deter terrestrial predators. Each enclosure is equipped with a straw-filled shed, two watering stations, and a feeding station, providing ad libitum access to the same mouse chow used in the laboratory (PicoLab Rodent Diet 20), as well as natural food sources found within the enclosures, such as berries, seeds, and insects. Eighteen WT, 10 Dectin-1^−/−^, 10 Dectin-1^+/−^, and 8 Card9^−/−^ female laboratory mice were housed in the enclosure for 6 to 7 weeks. Five WT, four Dectin-1^−/−^, five Dectin-1^+/−^, three Card9^−/−^, and eight Card9^+/−^ matched littermates were kept in the institutional vivarium for comparison. Longworth traps baited with chow were used to capture mice after release, with all rewilded mice captured for terminal analyses and all laboratory control mice recovered. Following fecal collection from all laboratory and rewilded mice, euthanasia was performed by CO_2_ asphyxiation, and blood, spleen, mLNs, femur, and intestinal tissue were harvested. Macroscopic and microscopic parasite analysis in multiple organs confirmed that mice in the outdoor enclosure were free from parasite infection during rewilding experiments.

Graphs displaying quantification of cell populations by flow cytometry display all mice for which samples and data were available, grouped by environmental condition (laboratory versus rewilded). Effects of wedge, sex, genotype, and caging were assessed by separating mice on the basis of these parameters and comparing across groups. None of these features were found to contribute to the variances in cell population numbers. scRNA-seq and microbiome analyses were performed on randomly selected WT mice within the same cohort. The animal work was conducted in accordance with approved protocols from the NYU Langone Institutional Animal Care and Use Committee (IACUC #IA16-0087 and #IA16-00864) and Princeton IACUC (#1982-17).

### Flow cytometry analysis

Whole blood was collected through cardiac puncture from mice and kept in a heparin-containing tube on ice. Femur was isolated from the surrounding tissue and cleaned with a surgical scalpel and paper towels to remove surrounding muscle and connective tissue. The BM content was flushed out and collected for further process. mLNs were isolated by careful dissection, and single-cell suspensions were prepared in 1 ml of fluorescence-activated cell sorting (FACS) buffer [phosphate-buffered saline (PBS), 2% fetal bovine serum (FBS), and 1 mM EDTA]. Whole spleens were isolated and bisected with a surgical scalpel, and 1-ml single-cell suspensions were prepared from half a spleen. Two hundred microliters of blood, 500 μl of a single-cell spleen suspension, and all BM cells were resuspended in 5 and 2.5 ml of 1× red blood cell (RBC) lysis buffer (BioLegend), respectively, for 10 min to lyse the RBCs. After two washes with cold PBS, the cells were signalized through 35-μm strainer with ice-cold FACS buffer. Single-cell suspensions of BM cells in FACS buffer were counted using an automatic cell counter with trypan blue (Countess 3, Thermo Fisher Scientific). For cell surface staining, the cells were incubated with antibodies at 4°C for 20 min and fixed for 20 min at room temperature. For cell surface phenotype analysis, anti-B220 (RA3-6B2, 1:150), anti-CD117 (2B8, 1:200), anti-CD138 (281-2, 1:800), anti-CD19 (1D3, 1:800), anti-CD1d (1B1, 1:200), anti-CD23 (B3B4, 1:200), anti-CD25 (PC61.5, 1:200), anti-CD38 (90, 1:300), anti-3e (500A2, 1:200), anti-CD44 (IM7, 1:300), anti-CD5 (53-7.3, 1:500), anti-CD69 (H1.2F3, 1:300), anti-CD93 (AA4.1, 1:300), anti-CD95 (Jo2, 1:200), anti-IgM (AF6-78, 1:500), anti-IgA (11-44-2, 1:200, anti-IgD (11-26c, 1:50), anti-IgG1 (A85-1, 1:500), anti-MHCII (M5/114.115.2, 1:200), and peanut agglutinin (1:400, Vector Laboratories) were used. Dead cells were excluded using Invitrogen Fixable Blue Dead Cell Stain Kit (Thermo Fisher Scientific). Flow cytometry data were acquired on the ZE5 cell analyzer (Bio-Rad) and analyzed on Flowjo v10.8.1.

### Immunofluorescence

The terminal ileum was fixed in 10% formalin and embedded in paraffin blocks. Paraffin embedded tissue sections (5 μm) were deparaffinized using xylene and rehydrated using a descending ethanol gradient (100, 95, 70%, pure dH_2_O). Antigen retrieval was conducted by heating slides in a pressure cooker (Electron Microscopy Sciences). Tissues were permeabilized with 1× PBS containing 0.1% Triton X-100 (Sigma-Aldrich) for 10 min at room temperature and washed three times with 1× PBS containing 0.01% Tween 20 (Sigma-Aldrich). Then, tissue sections were blocked in 1× PBS containing 5% bovine serum albumin for 1 hour at 37°C followed by incubation at 4°C overnight in 1× PBS containing 5% bovine serum albumin and primary antibodies against IgM (1:200, Arigo Biolabotories) and CD138 (281-2, 1:100). After washing the slides three times with 1× PBS, the sections were incubated with anti-goat IgG Fab2 Alexa Fluor 568 (1:500, Thermo Fisher Scientific) or anti-rat IgG Fab2 Alexa Fluor 488 (1:500, Thermo Fisher Scientific), respectively. Nuclei were visualized using ProLong Glass Antifade Mountant with NucBlue Stain (Thermo Fisher Scientific). Images were acquired with Zeiss LSM710.

### Isolation of mouse lamina propria cells

Lamina propria cells were isolated following a previously described protocol (*34*). In short, small intestine tissues were cut open, washed with PBS, and had fat removed. The tissues were incubated first with 20 ml of Hanks’ balanced salt solution (HBSS; Gibco) with 2% Hepes, 1% sodium pyruvate, 5 mM EDTA, and 1 mM dl-dithiothreitol (Sigma-Aldrich) for 15 min at 37°C with agitation at 220 rpm. They were then incubated in a fresh 20 ml of HBSS containing 2% Hepes, 1% sodium pyruvate, and 5 mM EDTA for an additional 10 min under the same conditions. The tissue bits were washed with HBSS, minced, and then enzymatically digested with collagenase (0.5 mg/ml; Sigma-Aldrich) and deoxyribonuclease I (0.01 mg/ml; Sigma-Aldrich) for 30 min at 37°C with agitation at 200 rpm. The digested solutions were passed through 70-μm nylon mesh (ELKO Filtering), and the isolated cells were resuspended in 40% Percoll (Sigma-Aldrich), layered onto 80% Percoll, and centrifuged without brake at room temperature at 1040 rcf for 22 min. The cells were collected from the interphase and washed with RPMI1640 (Corning) containing 10% FBS and used as lamina propria cells.

### Pathogen screen

Randomly selected rewilded mice from every genotype underwent screening for the presence of infectious agents using the EZ-Spot Assessment Plus Multiplexed Fluorometric Immunoassay and PCR Infectious Agent Testing Surveillance Plus Panel (Charles River Laboratories). Dried blood, feces, and body swabs were collected following submission guidelines.

### Single-cell RNA sequencing

Small intestine cells in lamina propria were harvested as previously described (*34*) from four male laboratory and four female rewild mice. Total cells from each group were pooled, and immune cells were sorted with CD45^+^ antibodies. The cells then were counted using a Bio-Rad TC20 automated cell counter. Single cells were then encapsulated into emulsion droplets using Chromium Controller (10x Genomics). scRNA-seq libraries were constructed using Chromium Single Cell 3′ v3.3 Reagent Kit (PN-1000268) according to the manufacturer’s protocol. Amplified cDNA was evaluated on an Agilent BioAnalyzer 2100 with a High Sensitivity DNA Kit (Agilent Technologies) and final libraries on an Agilent TapeStation 4200 using High Sensitivity D1000 ScreenTape (Agilent Technologies). Individual libraries were diluted to a concentration of 2 nM and combined into pools for sequencing. The pooled libraries were sequenced using 100-cycle run kits [28 base pairs (bp) for Read 1, 8 bp for Index 1, and 91 bp for Read 2] on the NovaSeq 6000 Sequencing System (Illumina).

### scRNA-seq data processing

The Cell Ranger’s pipeline version 7.1.0 was used to demultiplex cellular barcodes and aligned reads against the mouse genome including introns (mm10 ensemble). Subsequent RNA-seq analysis was done with Seurat version 5.0.3 on R version 4.2.1 with the filtered RNA and HTO featured counts. Ambient RNA contamination was removed using SoupX, which automatically calculates the contamination fraction for the estimation and removal of cell-free mRNA contamination in droplet. Doublets were identified and removed using scDblFinder with standard parameters. Cells with more than 10% mitochondrial DNA, fewer than 300 feature genes, or more than 4000 feature genes were excluded during initial quality control. RNA normalization was performed using regularized negative binomial regression via the SCTransform function. Samples were integrated using the Harmony package. PCoA was performed using Harmony embeddings, and the Louvain algorithm determined unsupervised clustering with 30 dimensions. Uniform Manifold Approximation and Projection representation based on totalVI dimension reduction of RNA was used to visualize data. Cell types were determined by a combination of unbiases clustering, canonical cell type marker signatures, and cell type annotation via the SingleR package with the ImmGenData open-source (expression) reference databases in the CellDex package. Differentially expressed genes between clusters and treatment groups were assessed using the Wilcoxon test with Benjamini-Hochberg *P* value adjustment. Gene ontology enrichment analysis was preformed using the clusterProfiler package using all three orthogonal ontologies using *P* and *q* value cutoffs of 0.5 on enrichment tests.

### BCR sequence reconstitution

BCR sequences were generated from scRNA-seq data using TRUST4 v1.14. The BCR sequence references were obtained from the following source: TRUST4 GitHub repository. The annotation of the consensus assembly was performed using the annot.fa files from the TRUST4 output.

### BCR gene assignments

The germline V(D)J gene was first annotated using IgBLAST v.1.20, in tandem with the IMGT/GENE-DB release 202113-2. The output generated by IgBLAST was then parsed with the assistance of Change-O v.1.3.0. To ensure data quality, we set up a series of requirements for each sequence. These included the presence of non-empty V and J gene annotations, the consistency of chain annotations, sequences with less than 10 noninformative “N” positions (non-A/T/G/C) in the entire sequence alignment, and a CDR3 that is devoid of N and has a nucleotide length divisible by 3. Any sequences that IgBLAST annotated as nonproductively rearranged were subsequently excluded from our analysis.

### Clonal lineage analysis

We inferred B cell clonal lineages from productively rearranged heavy chain sequences using hierarchical clustering with single linkage. Initially, sequences were partitioned according to common V and J gene annotations and CDR3 lengths. Using the SHazaM R package, we generated the nearest-neighbor distance distribution with the distToNearest function. We then calculated nearest neighbor distances independently on the basis of the environment and calculated the nearest neighbor distances across subjects to initialize the Gaussian fit parameters of the inter-clonal nearest-neighbor distance distribution to estimate the appropriate threshold for clonal assignments, 0.03.

Post clonal clustering, Dowser was used to reconstruct clonal germlines. We reconstructed full-length clonal consensus germline sequences for each clone, masking D-segment and N/P regions with Ns, and resolving any ambiguous gene assignments by majority rule. Within each clone, we collapsed duplicate IMGT-aligned V(D)J sequences from bulk sequencing, excluding duplicates derived from different B cell compartments or isotypes. We constructed clonal lineage trees using IgPhyML and graphed them as ggtrees.

### BCR characterization

Mutational profiling was performed with Shazam, which included replacement and silent mutations throughout the entire sequence. This enabled us to compare mutational burden within a cell and quantify median mutational frequency within clones. Abundance and diversity were quantified using the delta of the bootstrap distributions between groups, facilitated by the Alazkam package. We visualized clones as networks with NAIR: Network Analysis of Immune Repertoire. In these networks, each node represents a BCR-containing cell, and each edge signifies a clonal relationship. The distance algorithm we used is a random walk variation based on clone identifiers.

### Mouse total IgG and IgM measurement

Whole blood was collected through cardiac puncture from mice and kept in a heparin-containing tube on ice. After centrifuging at 400 rcf for 5 min, the plasma was collected from the supernatant for further assays. Total IgG and IgM concentrations from laboratory and rewilded mice were measured using mouse IgG enzyme-linked immunosorbent assay (ELISA) antibody pair kits and mouse IgM ELISA antibody pair kits (STEMCELL Technologies) according to the manufacturer’s instructions. Each sample was assayed in duplicate. Standard curves for each antibody isotype were generated using the reference Ig concentrations provided by the manufacturer. Total IgG and IgM concentrations were calculated on the basis of these readings, with reference curves determined from the kit standards. Samples with duplicate coefficients of variation greater than 10% in absorbance readings were discarded and rerun on another plate.

### IgG-seq and IgM-seq on paired plasma and stool samples

Stools from laboratory and rewilded mice were suspended in buffer (1% w/v bovine serum albumin in PBS) and mechanically disrupted using sterile pipette tips, followed by homogenization through repeated pipetting using wide-bore pipette tips. After centrifugation at 50 rcf for 1 min to pellet nonbacterial food particles, the supernatant was filtered through a 40-mm filter into a new sterile tube and washed by centrifugation at 8000 rcf for 3 min. One-tenth of this suspension was used to quantify optical density at 600 nm for normalization of input samples. The samples were divided for IgG-seq and IgM-seq analyses. Both sets of samples were incubated in 1:50 plasma for 30 min at 4°C. Afterward, the samples were resuspended in staining mixtures containing SYTO-62 (1:500, Invitrogen); anti-IgM (II/41, 1:50) for IgM-seq samples; and anti-IgG1 phycoerythrin (PE) (A85-1, 1:300), anti-IgG2ab PE (X57, 1:20), anti-IgG3 PE (B10, 1:1500) for IgG-seq samples. All staining procedures were conducted for 15 min at 4°C. Following washing steps, the samples underwent magnetic column–based fractionation, DNA extraction, 16*S* rRNA amplification, pooling, and sequencing, as described previously.

### Quantification and statistical analysis

Statistical parameters including the definition of central value and the exact number (*n*) of mice per group are annotated in the corresponding figure legend. Data presented in graphs and bar plots are shown as means ± SD. Statistical analysis was performed with GraphPad Prism version 9.2 for Mac (GraphPad). Differences between two groups were assessed using an unpaired two-tailed Student’s *t* test. *P* values are indicated in the figures, with “ns” denoting nonsignificant differences.
