# Supplementary material for: Rewilding catalyzes maturation of the humoral immune system
Source: Sci Adv. 2025 Mar 7;11(10):eads2364. doi: 10.1126/sciadv.ads2364 (PMC11887799; doi:10.1126/sciadv.ads2364)
Supplement: Supplementary file 1 — Figs. S1 to S6 Tables S1 and S2 [file sciadv.ads2364_sm.pdf]

Science Advances
AAAS

Supplementary Materials for

Rewilding catalyzes maturation of the humoral immune system

Ying-Han Chen *et al.*

Corresponding author: Ken Cadwell, ken.cadwell@Pennmedicine.upenn.edu;
Sergei B. Koralov, sergei.koralov@nyulangone.org; Ying-Han Chen, ychen@ibms.sinica.edu.tw

Sci. Adv. **11**, eads2364 (2025)
DOI: 10.1126/sciadv.ads2364

This PDF file includes:

Figs. S1 to S6
Tables S1 and S2

Fig. S1

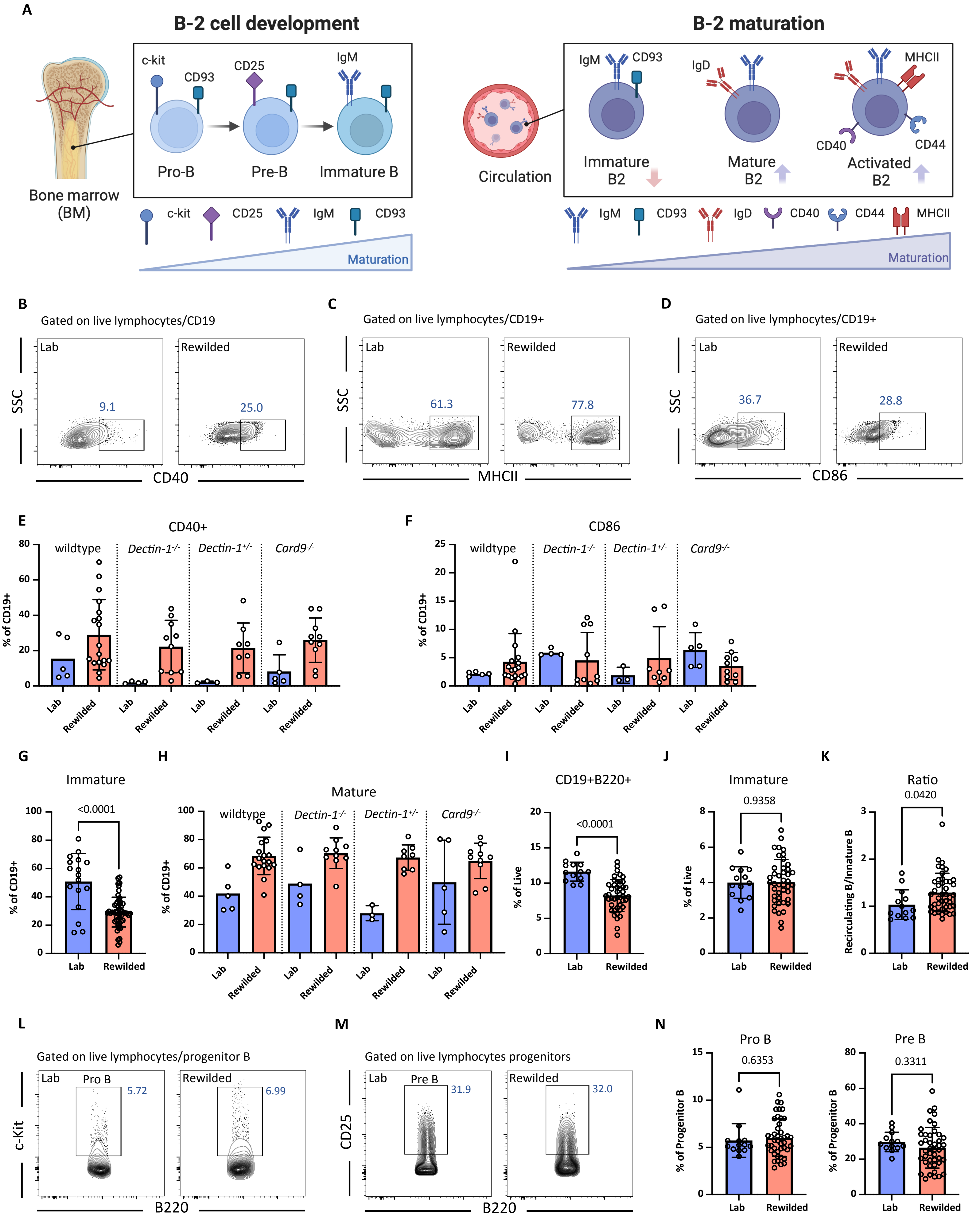
A
B-2 cell development
c-kit
CD93
CD25
IgM
Pro-B
Pre-B
Immature B
Bone marrow (BM)
Maturation
B-2 maturation
IgM
CD93
IgD
MHCII
Immature B2
Mature B2
CD40
CD44
Activated B2
Circulation
Maturation
B
Gated on live lymphocytes/CD19
Lab
Rewilded
9.1
25.0
SSC
CD40
C
Gated on live lymphocytes/CD19+
61.3
77.8
MHCII
D
36.7
28.8
CD86
E
CD40+
wildtype
Dectin-1-/-
Dectin-1+/-
Card9-/-
% of CD19+
F
CD86
G
Immature
<0.0001
H
Mature
I
CD19+B220+
% of Live
J
Immature
0.9358
K
Ratio
0.0420
Recirculating B/Innature B
L
Gated on live lymphocytes/progenitor B
Pro B
5.72
6.99
c-Kit
B220
M
Gated on live lymphocytes progenitors
Pre B
31.9
32.0
CD25
N
Pro B
0.6353
Pre B
0.3311
% of Progenitor B

Fig. S1: Alterations in B cell populations in blood and bone marrow associated with rewilding (supplement to Figure 1).

(A) Schematic of B cell states of maturation and activation with surface markers used in this study. **(B to D)** Representative flow cytometry plots depicting gating strategy for CD40+, MCHII+, and CD86 B cells in peripheral blood from laboratory and rewilded mice. **(E)** Quantification of frequency of peripheral CD93+ immature B cells from figure 1 (E). **(F)** Frequency of B cells (CD19+B220+) in the bone marrow. **(G)** Quantification of frequency of immature B cells in bone marrow from figure 1 (j). **(H)** Ratio of recirculating fraction to immature B cells in bone marrow. **(I and J)** Representative flow cytometry plots depicting gating strategy used to identify pro and pre B cells in the bone marrow. A subpopulation of B220+IgM- progenitor B cells (figure 1J) was characterized as pro and pre B by c-Kit and CD25 respectively. **(K)** Frequency of subpopulations of B220+IgM- progenitor B cells (pro B and pre B) in the bone marrow. N = 18 laboratory and 54 rewilded mice. Dots in bar graphs correspond to individual mice. Mean and SD are shown. Indicated p values by two-tailed Student's t test between groups.

Fig. S2

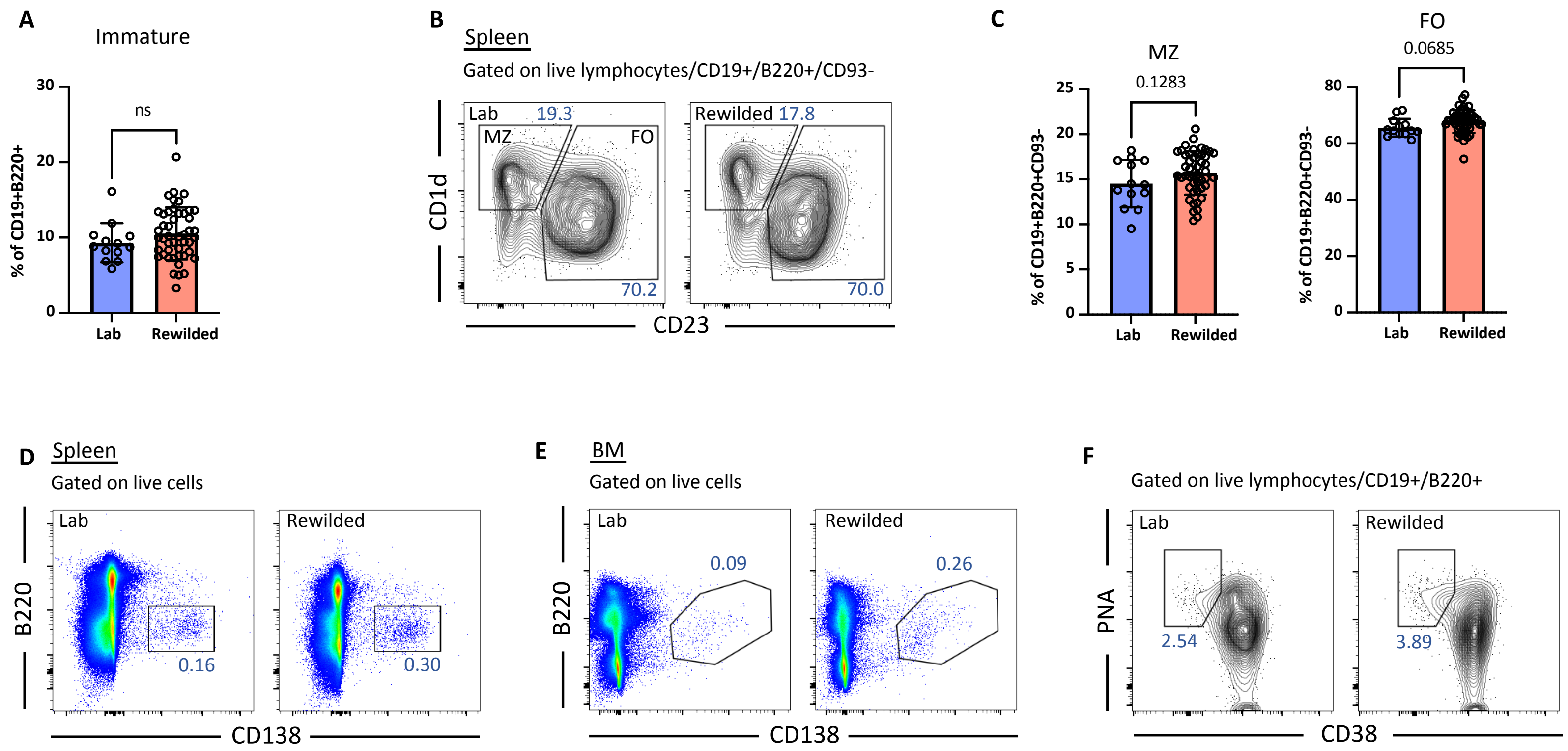

A
Immature
% of CD19+B220+
ns
Lab
Rewilded
B
Spleen
Gated on live lymphocytes/CD19+/B220+/CD93-
Lab 19.3
MZ
FO
70.2
Rewilded 17.8
70.0
CD1d
CD23
C
MZ
0.1283
% of CD19+B220+CD93-
FO
0.0685
D
Spleen
Gated on live cells
Lab
Rewilded
0.16
0.30
B220
CD138
E
BM
Gated on live cells
0.09
0.26
F
Gated on live lymphocytes/CD19+/B220+
2.54
3.89
PNA
CD38

Fig. S2: Germinal center responses in rewilded mice (supplement to Figure 2).

(A) Quantification of frequency of mature B cells in the spleen of laboratory and rewilded mice. **(B)** Representative flow cytometry plots depicting gating strategy for marginal zone (MZ) and follicular (FO) B cells in the spleen from laboratory and rewilded mice. MZ and FO B cells were gated on CD19+B220+CD93- B cells and identified by CD23 and CD1d. **(C)** Frequency of MZ and FO B cells from (B). **(D and E)** Representative flow cytometry plots of plasma cells (CD138+) in the spleen and bone marrow from laboratory and rewilded mice. **(F)** Representative flow cytometry plots for germinal center B cells in the mLN from laboratory and rewilded mice. Germinal center B cells were gated on CD19+B220+ B cells and identified by CD38 and PNA. Dots in bar graphs correspond to individual mice. Mean and SD are shown. Indicated p values by two-tailed Student's t test between groups.

Fig. S3

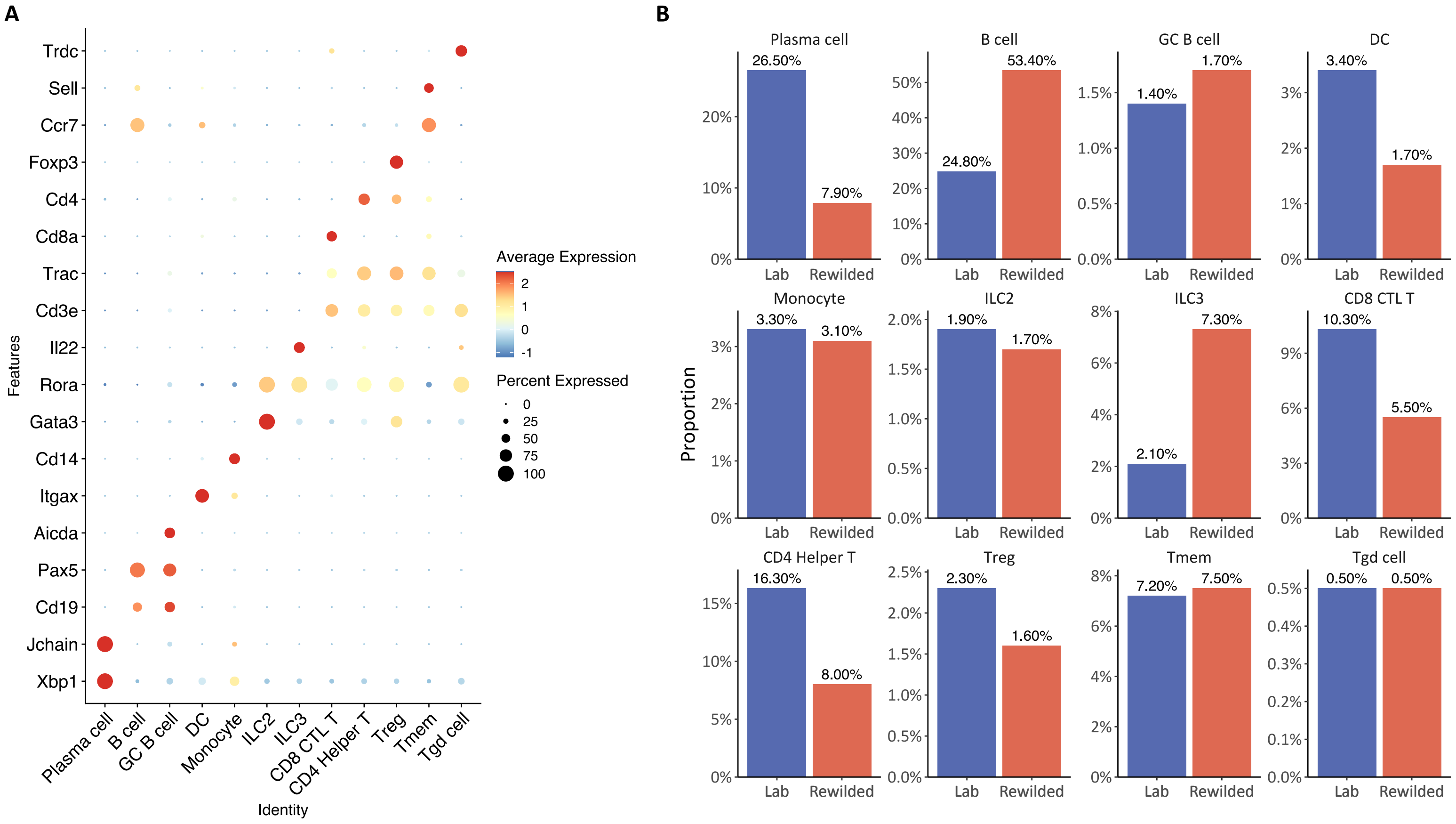

A
Trdc
Sell
Ccr7
Foxp3
Cd4
Cd8a
Trac
Cd3e
Il22
Rora
Gata3
Cd14
Itgax
Aicda
Pax5
Cd19
Jchain
Xbp1
Features
Plasma cell
B cell
GC B cell
DC
Monocyte
ILC2
ILC3
CD8 CTL T
CD4 Helper T
Treg
Tmem
Tgd cell
Identity
Average Expression
2
1
0
-1
Percent Expressed
0
25
50
75
100
B
Proportion
Plasma cell
26.50%
7.90%
B cell
24.80%
53.40%
GC B cell
1.40%
1.70%
DC
3.40%
1.70%
Monocyte
3.30%
3.10%
ILC2
1.90%
1.70%
ILC3
2.10%
7.30%
CD8 CTL T
10.30%
5.50%
CD4 Helper T
16.30%
8.00%
Treg
2.30%
1.60%
Tmem
7.20%
7.50%
Tgd cell
0.50%
0.50%
Lab
Rewilded

Fig. S3: scRNA-seq analysis of intestinal immune cells from rewilded and laboratory mice (supplement to Figure 3).

(A) Dot plot for gene expression of each delineated clusters. **(B)** Proportion of various immune cells in the small intestine of laboratory and rewilded mice.

Fig. S4

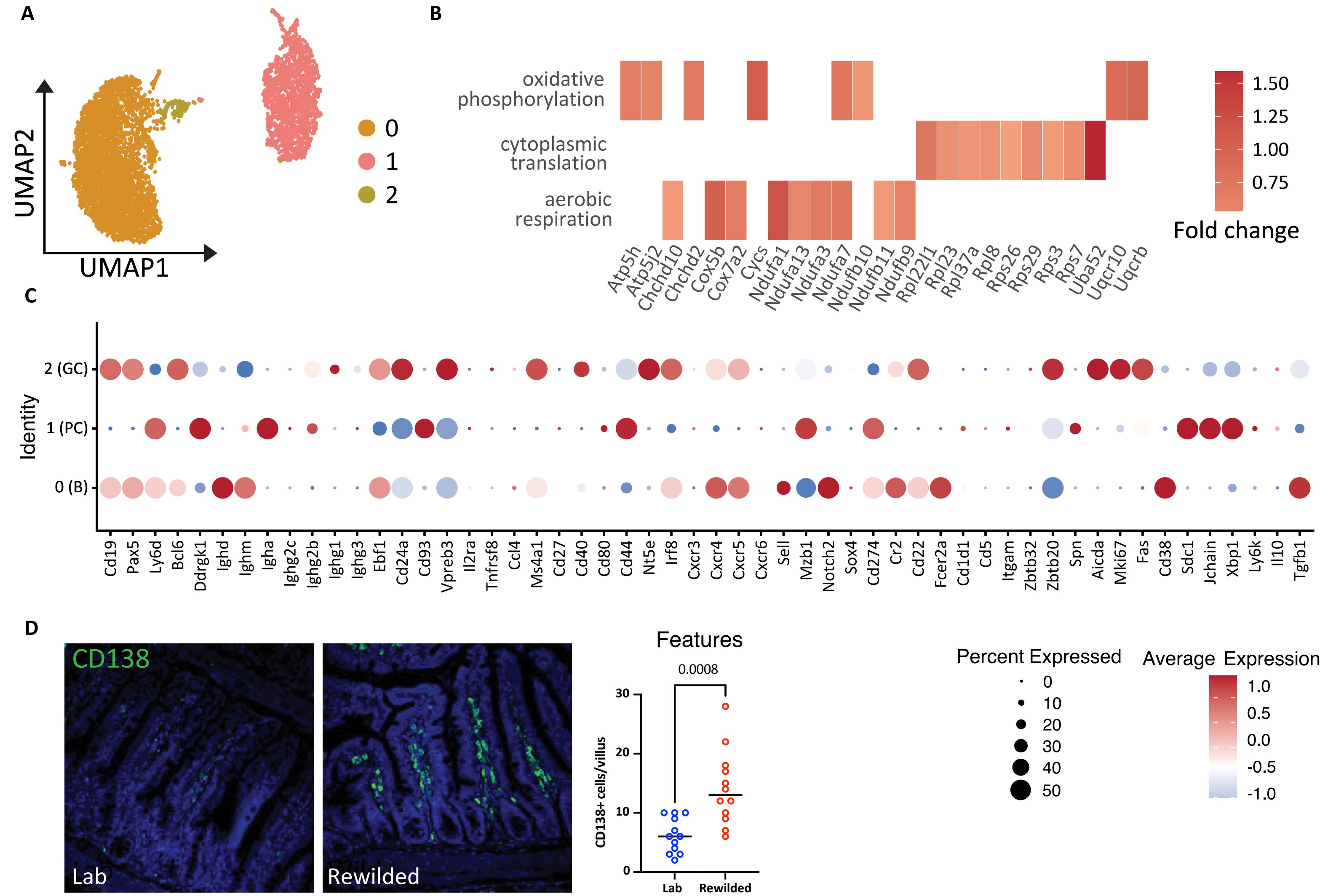

A
UMAP2
UMAP1
0
1
2
B
oxidative phosphorylation
cytoplasmic translation
aerobic respiration
Atp5h Atp5j2 Chchd10 Chchd2 Cox5b Cox7a2 Cycs Ndufa1 Ndufa13 Ndufa3 Ndufa7 Ndufb10 Ndufb11 Ndufb9 Rpl22l1 Rpl23 Rpl37a Rpl8 Rps26 Rps29 Rps3 Rps7 Uba52 Uqcr10 Uqcrb
1.50
1.25
1.00
0.75
Fold change
C
Identity
2 (GC)
1 (PC)
0 (B)
Cd19 Pax5 Ly6d Bcl6 Ddrgk1 Ighd Ighm Igha Ighg2c Ighg2b Ighg1 Ighg3 Ebf1 Cd24a Cd93 Vpreb3 Il2ra Tnfrsf8 Ccl4 Ms4a1 Cd27 Cd40 Cd80 Cd44 Nt5e Irf8 Cxcr3 Cxcr4 Cxcr5 Cxcr6 Sell Mzb1 Notch2 Sox4 Cd274 Cr2 Cd22 Fcer2a Cd1d1 Cd5 Itgam Zbtb32 Zbtb20 Spn Aicda Mki67 Fas Cd38 Sdc1 Jchain Xbp1 Ly6k Il10 Tgfb1
D
CD138
Lab
Rewilded
Features
0.0008
CD138+ cells/villus
30
20
10
0
Lab
Rewilded
Percent Expressed
0
10
20
30
40
50
Average Expression
1.0
0.5
0.0
-0.5
-1.0

Fig. S4: Analyses of B cell clusters from scRNA-seq of small intestine from rewilded and laboratory mice (supplement to Figure 4).

(A) UMAP of subsets of B cell-lineage clusters. Different clusters indicated by colors legend. **(B)** Gene fold-change differences of top three pathways of up-regulated genes from mature B cells of rewilded mice. **(C)** Dot plot for marker B cell genes of each delineated clusters. **(D)** Confocal images of ileum sections immunostained with anti-CD138 antibodies and counterstained with DAPI. The imaging is representative of three mice. CD138+ cells were quantified by selecting four random villi from three individual mice per condition. Mean and SD are shown. Indicated p values by two-tailed Student's t test between groups.

Fig. S5

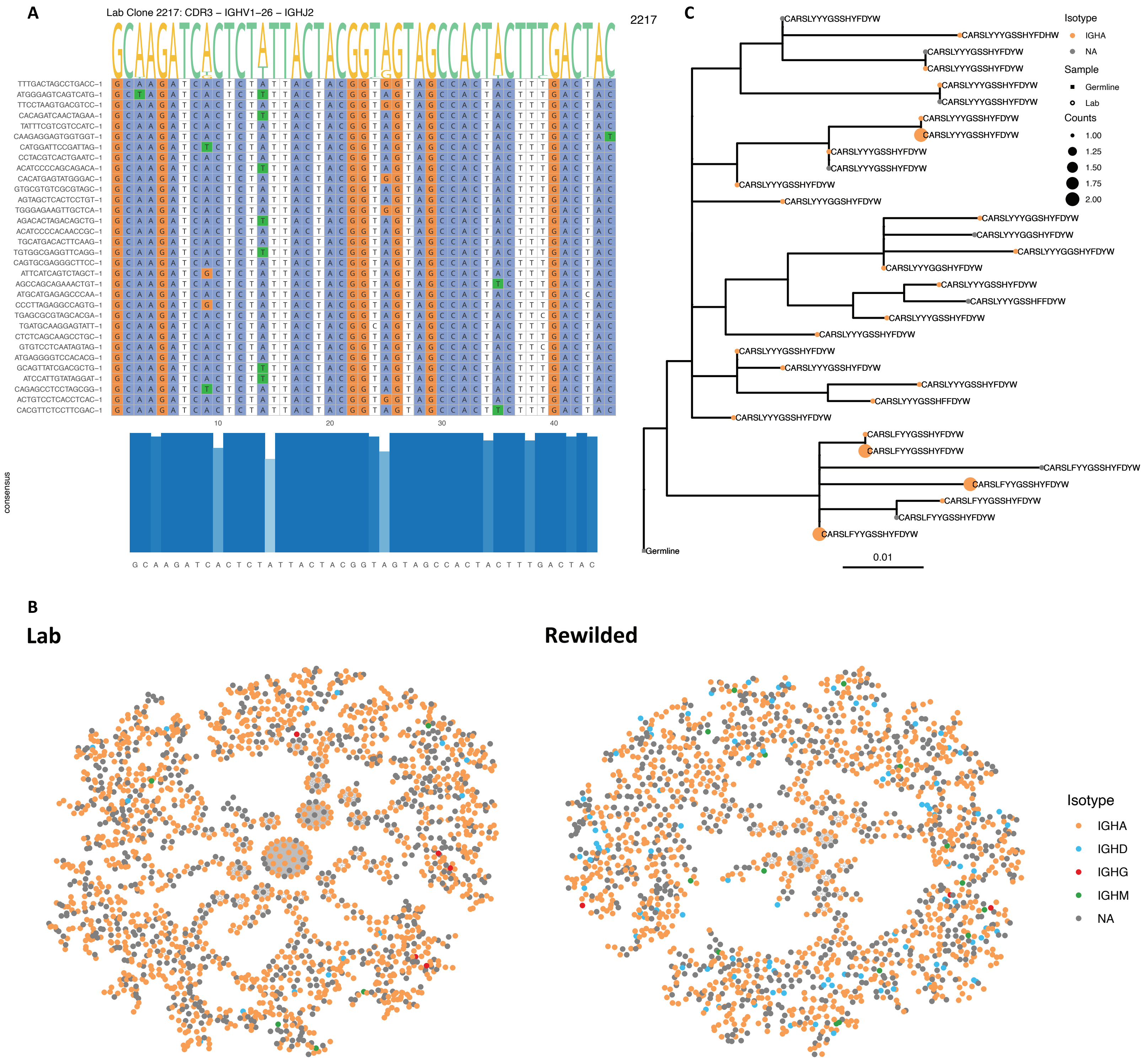

A
Lab Clone 2217: CDR3 – IGHV1–26 – IGHJ2
2217
consensus
10
20
30
40
G C A A G A T C A C T C T A T T A C T A C G G T A G T A G C C A C T A C T T T G A C T A C
C
CARSLYYYGSSHYFDYW
CARSLYYYGSSHYFDHW
CARSLYYYGGSHYFDYW
CARSLYYYGSSHFFDYW
CARSLYYGSSHYFDYW
CARSLYYYGGSHFFDYW
CARSLFYYGSSHYFDYW
Germline
0.01
Isotype
IGHA
NA
Sample
Germline
Lab
Counts
1.00
1.25
1.50
1.75
2.00
B
Lab
Rewilded
Isotype
IGHA
IGHD
IGHG
IGHM
NA

Fig. S5: Rewilding increases BCR diversity and plasma cell mutation frequency (supplement to Figure 4).

(A) Junction region sequences of the laboratory-expanded clone 2217. Each row signifies a BCR junction sequence per cell, with individual cells identified by cellular barcodes on the left y-axis. The size of the amino acid in the top axis represents the frequency of nucleotides per location. The bottom bar graph illustrates the mutational frequency per location. **(B)** Clonal BCR network divided between laboratory and rewilded mice. Each dot, or node, represents an individual cell, while each connecting line, or edge, signifies a clonal relationship between nodes. The color of each node corresponds to the isotype of the BCR-containing cell. **(C)** Evolutionary lineage tree for the laboratory-expanded clone 2217. Each dot represents a BCR belonging to the expanded clone 2217, with its color indicating the BCR's isotype. The size of the dot signifies the frequency of the exact sequence found in cells. The distance between dots represents the number of steps between the complete BCR sequences, with the scale provided at the bottom.

Fig. S6

A

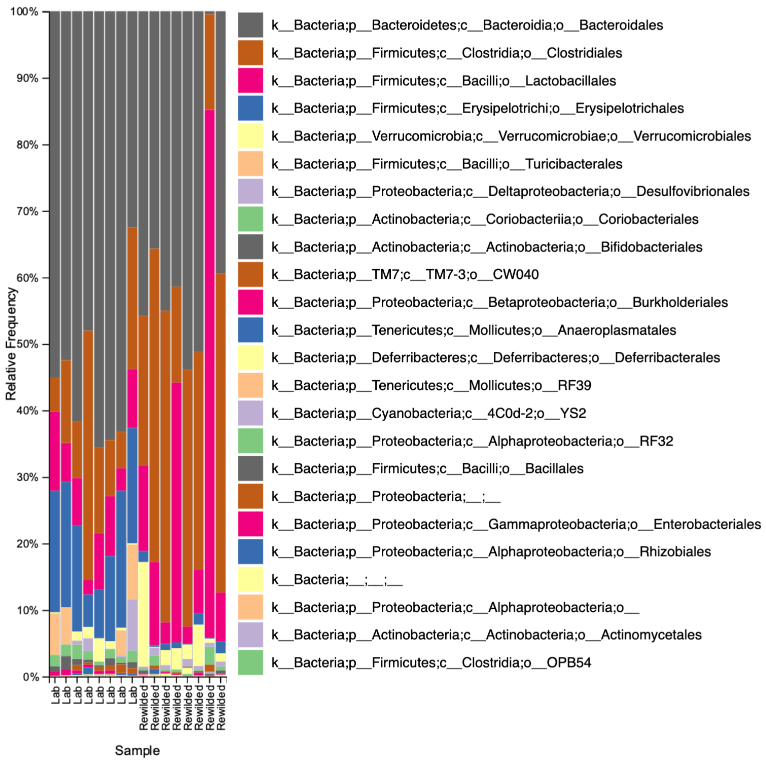

B

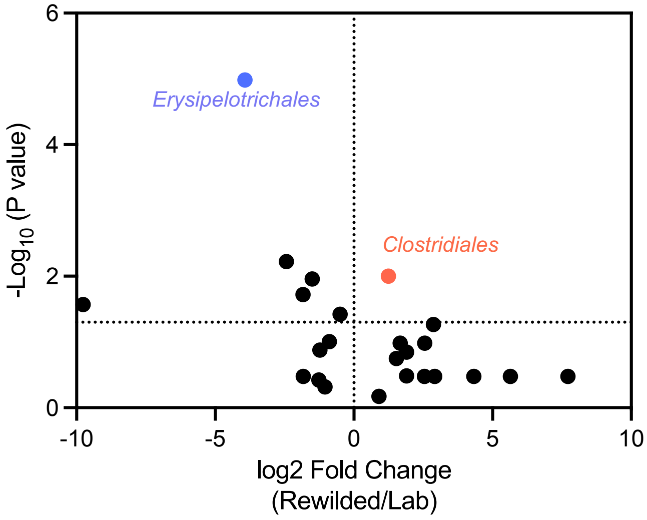

C

Taxa with higher **IgG** scores in **rewilded** mice

ASV#	Nearest identity via BLAST	Identity (%)
1	Anaerobium acetethylicum	91.30
2	Flintibacter butyricus	92.91
3	Anaerosporobacter mobilis	94.48
4	Lactobacillus faecis	100.00
5	Lactobacillus faecis	99.60
6	Intestinibacillus massiliensis	98.41
7	Kineothrix alysoides	95.65
8	Christensenella timonensis	90.87
9	Flintibacter butyricus	92.91
10	Lactobacillus reuteri	100.00
11	Lachnoclostridium pacaense	97.23
12	Clostridium alkalicellulosi	86.56

Taxa with higher **IgM** scores in **rewilded** mice

ASV#	Nearest identity via BLAST	Identity (%)
1	Flintibacter butyricus	100.00
2	Flintibacter butyricus	98.024
3	Kineothrix alysoides	96.443
4	Anaerotruncus colihominis	94.444
5	Murimonas intestini	92.885
6	Roseburia intestinalis	92.885
7	Frisingicoccus caecimuris	94.862
8	Desulfovibrio desulfuricans	95.652
9	Muribaculum intestinale	94.466
10	Bacteroides xylanolyticus	94.862
11	Eubacterium siraeum	96.443

D

Taxa with higher **IgG** scores in **lab** mice

ASV#	Nearest identity via BLAST	Identity (%)
1	Clostridium xylanolyticum	97.62
2	Eisenbergiella massiliensis	94.09
3	Lachnoclostridium pacaense	95.25
4	Akkermansia muciniphila	100.00
5	Faecalimonas umbilicata	94.07
6	Clostridium amygdalinum	94.46
7	Muricomes intestini	97.62
8	Eubacterium contortum	96.83
9	Murimonas intestini	95.25
10	Anaerobium acetethylicum	94.46
11	Muribaculum intestinale	88.49

Taxa with higher **IgM** scores in **lab** mice

ASV#	Nearest identity via BLAST	Identity (%)
1	Muribaculum intestinale	95.635

Fig. S6: Microbiome analyses of rewilded mice (supplement to Figure 5).

(A) Relative abundance of order level taxa that constitute the fecal microbial community of laboratory and rewilded mice as determined by 16S sequencing. N = 8 laboratory and 8 rewilded mice. **(B)** Volcano plot comparing rewilded versus lab mice gut microbiome. Statistical significance calculated with the Welch's t-test. **(C)** Taxa with greater IgG and IgM scores in rewilded mice. **(D)** Taxa with greater IgG and IgM scores in laboratory mice. ASV: amplicon sequence variant.

Serology	Wild
MFIA SEND	0/10 (0%)
MFIA PVM	0/10 (0%)
MFIA MHV	0/10 (0%)
MFIA MVM	0/10 (0%)
MFIA MPV1	0/10 (0%)
MFIA MPV2	0/10 (0%)
MFIA NS1	0/10 (0%)
MFIA MNV	0/10 (0%)
MFIA GDVII	0/10 (0%)
MFIA REO	0/10 (0%)
MFIA EDIM	0/10 (0%)
(ROTAA)	0/10 (0%)
MFIA LCMV	0/10 (0%)
MFIA ECTRO	0/10 (0%)
MFIA MAV 1 & 2	0/10 (0%)
MFIA MCMV	0/10 (0%)
MFIA K	0/10 (0%)
MFIA MTLV	0/10 (0%)
MFIA POLY	0/10 (0%)
MFIA HANT	0/10 (0%)
MFIA MPUL	0/10 (0%)
MFIA ECUN	0/10 (0%)
MFIA CARB	0/10 (0%)
MFIA PHV	0/10 (0%)
MFIA LDV	0/10 (0%)
MFIA Anti-Ig (Positive Ctrl)	100%

Table S1. Rewilded Mice Are Seronegative for Common Mouse Pathogens.

Multiplexed Fluorometric ImmunoAssay results from serum of rewilded mice (Animal Health Diagnostic Services, MFIA Mouse Assessment Plus Profile, Charles River). N = 10 rewilded mice.

Infectious disease PCR	Wild
MHV PCR	0/10 (0%)
Mouse Parvovirus (MPV/MVM) P	0/10 (0%)
MRV (EDIM)	0/10 (0%)
PVM	0/10 (0%)
SEND	0/10 (0%)
TMEV/GDVII	0/10 (0%)
Beta Strep Grp A	0/10 (0%)
Beta Strep Grp B	0/10 (0%)
Beta Strep Grp C	0/10 (0%)
Beta Strep Grp G	0/10 (0%)
B. bronchiseptica	0/10 (0%)
C. kutscheri	0/10 (0%)
C. piliforme	0/10 (0%)
C. rodentium	0/10 (0%)
P. pneumotropica-Heyl	0/10 (0%)
P. pneumotropica-Jawetz	0/10 (0%)
Ps. Aeruginosa	0/10 (0%)
K. pneumoniae	1/10 (10%)
K. oxytoca	1/10 (10%)
Salmonella Genus	0/10 (0%)
S. aureus	1/10 (10%)
S. moniliformis	0/10 (0%)
Entamoeba	0/10 (0%)
Giardia	0/10 (0%)
Mite	0/10 (0%)
Pinworm	0/10 (0%)
Spironucleus muris	0/10 (0%)
M. pulmonis	0/10 (0%)

Table S2. Rewilded Mice Are Negative for Most Agents Detected by PCR Screening. TaqMan PCR testing results from feces of rewilded mice (Animal Health Diagnostic Services, Surveillance Plus PRIA Panel, Charles River). N = 10 rewilded mice.
